# Endovascular repair of abdominal aortic aneurysm-related type II endoleak: a multicenter study on the possibility of further intervention

**DOI:** 10.3389/fcvm.2025.1450942

**Published:** 2025-04-17

**Authors:** E. Erdemutu, Chongbin Zhou, Ming Ma, Liqiang Hu, Jisiguleng Wu, Xiangchen Dai, Zhanfeng Gao

**Affiliations:** ^1^ Department of Vascular Surgery, Tianjin Medical University General Hospital, Tianjin, China; ^2^ Department of Vascular Surgery, Affiliated Hospital of Inner Mongolia Medical University, Hohhot, China; ^3^ Department of Vascular Surgery, Hohhot First Hospital, Hohhot, China; ^4^ Department of Vascular Surgery, Shanxi Provincial People’s Hospital, Taiyuan, China

**Keywords:** abdominal aortic aneurysm, type II endoleak, multicenter study, endovascular repair (EVAR), retrospective analysis

## Abstract

**Background:**

We aimed to analyze the risk factors associated with Type II endoleak (T2EL) requiring reintervention after endovascular aneurysm repair (EVAR) for multicenter abdominal aortic aneurysms.

**Methods:**

A retrospective analysis was conducted on data from 614 patients with abdominal aortic aneurysms who underwent elective EVAR at three centers (Tianjin Medical University General Hospital, Affiliated Hospital of Inner Mongolia Medical University, Shanxi Provincial People's Hospital) from January 2017 to December 2021. After applying exclusion criteria, 375 patients were included in the study, with 50 patients in the T2EL-related reintervention group and 325 patients in the non-T2EL group. Single-factor and multiple-factor logistic analyses were used to identify high-risk factors, and ROC curve analysis was performed to determine the risk thresholds for mesenteric artery diameter, number of lumbar arteries, maximum aneurysm diameter, and proportion of intraluminal thrombus volume.

**Results:**

The rate of T2EL-related reintervention among the 375 patients was 13.33% (50/375). Single-factor analysis indicated that age, hypertension, maximum aneurysm diameter, proportion of intraluminal thrombus, diameter of inferior mesenteric artery (IMA), and number of patent lumbar arteries (LA) were risk factors for T2EL-related reintervention. Multiple-factor logistic analysis identified maximum aneurysm diameter, proportion of thrombus, IMA diameter, and number of patent LA as the main influencing factors for T2EL-related reintervention after EVAR. Significant risk factors for reintervention were maximum aneurysm diameter (OR = 1.043, 95% CI 1.015–1.072, *P* = 0.002), IMA diameter (OR = 3.901, 95% CI 1.116–13.632, *P* = 0.033), and number of LA (OR = 2.584, 95% CI 1.722–3.769, *P* < 0.001). A significant protective factor for reintervention was thrombus proportion (OR = 0.895, 95% CI 0.864–0.927, *P* < 0.001). ROC curve analysis showed that the risk thresholds for reintervention were an IMA diameter of 2.95 mm, intraluminal thrombus volume proportion <42.5%, number of LA ≤5.5, and aneurysm diameter of 53.55 mm.

**Conclusion:**

Cases with identified risk factors are considered to have a higher risk of T2EL-related reintervention after EVAR. Exceeding the new risk thresholds may indicate a higher likelihood of T2EL-related reintervention after EVAR.

## Introduction

Abdominal aortic aneurysm (AAA), a prevalent condition in vascular surgery, is characterized by a localized dilation of the abdominal aorta that exceeds 50% of the normal arterial diameter. Based on diagnostic criteria from both domestic and international sources (1, 2), an AAA with a diameter greater than 30 mm is diagnosed as an abdominal aortic aneurysm. The incidence of AAA has been increasing year by year. Currently, the preferred treatment for AAA is endovascular aneurysm repair (EVAR), which offers advantages such as minimal trauma, faster postoperative recovery, shorter hospital stay, and lower occurrence of perioperative complications. However, 30%–50% of AAA patients have anatomical features that may fall outside the standard indications for use (IFU) of EVAR devices, requiring advanced techniques or customized solutions (3). This means that when undergoing endovascular aneurysm repair (EVAR), these patients often require more advanced medical technology and equipment. Although these conditions do not necessarily exceed the guidelines outlined in the Instructions for Use (IFU) of EVAR devices, they do increase the complexity and technical demands of the procedure. As such, they represent one of the key challenges currently facing EVAR technology. In recent years, significant advancements have been made in endovascular treatment, encompassing both the refinement of surgical techniques and conceptual approaches, as well as the progressive optimization of associated medical devices. The proportion of EVAR performed on AAA patients with anatomical abnormalities has been increasing. Nevertheless, the advantages of EVAR have diminished over time due to a series of complications such as endoleaks. Endoleak, first defined by White et al. in 1996 (4), refers to the persistent blood flow within the aneurysm sac despite endovascular repair. This phenomenon has been systematically classified into five distinct types (I-V) based on its anatomical origin (5). Among these, Type II endoleak (T2EL), which results from retrograde flow through collateral vessels (such as lumbar or inferior mesenteric arteries) that remain patent after endograft placement, represents the most frequently encountered endoleak subtype following endovascular aortic repair (EVAR) (6). An observational study and recent meta-analysis have shown that the incidence of T2EL ranges from 10.2%–29.0% (7). However, not all T2ELs require reintervention. According to the latest guidelines from the United States (8) and Europe (9), isolated T2ELs without sac expansion should be treated conservatively, while intervention is recommended when sac expansion exceeds 10 mm (10). In conclusion, while endovascular aortic repair (EVAR) demonstrates substantial clinical advantages over traditional open surgical repair, it is associated with inherent limitations, particularly regarding Type II endoleak (T2EL) management. The current clinical challenge lies in the inability to reliably predict the necessity for T2EL-related reinterventions based on preoperative imaging and patient characteristics, highlighting a critical area for future research and technological advancement in endovascular therapy. Therefore, the purpose of this study is to retrospectively analyze clinical data from multicenter EVAR procedures for AAA and assess the risk rate of T2EL-related reinterventions after EVAR.

## Patients and methods

### Inclusion criteria and follow-up

Patient data of abdominal aortic aneurysm (AAA) cases treated with successful endovascular aneurysm repair (EVAR) in three vascular surgery centers in northern China (Tianjin Medical University General Hospital, Affiliated Hospital of Inner Mongolia Medical University, and Shanxi Provincial People's Hospital) between January 2017 and December 2021 were collected. The study was conducted from October to December 2022. Cases that did not meet the inclusion criteria were excluded based on the following exclusion criteria: (1) Open-window EVAR (*n* = 49); (2) Pre-embolization during EVAR (*n* = 44); (3) Ruptured abdominal aortic aneurysm (*n* = 27); (4) Incomplete medical records (*n* = 98); (5) Concurrent thoracic aortic diseases (*n* = 21).

After exclusions, a total of 376 patients were included in the analysis, divided into two groups: T2 endoleak (T2EL) reintervention group (*n* = 50) and non-T2EL group (*n* = 375). All patients underwent color Doppler ultrasound follow-up at 3 and 6 months postoperatively, followed by CT angiography (CTA) at 12 months and annually thereafter to monitor the need for T2EL-related reinterventions. If patients experienced any discomfort or recurrent symptoms, they were advised to seek immediate medical attention. The follow-up endpoint was the latest imaging examination or reintervention due to T2EL. The analysis showed that the average time from EVAR implantation to T2EL intervention was 16.98 months.

The mean implanted neck diameter was 25.16 mm, and the mean neck length was 30.96 mm. These measurements were consistent with the standard indications for use (IFU) of the EVAR devices employed in this study. The devices used included Medtronic Endurant (*n* = 187), Cook Zenith (*n* = 123), and Gore Excluder (*n* = 65). Device-specific outcomes were analyzed to assess potential relationships with endoleak rates.

Reintervention was indicated in cases of:

1. Aneurysm sac expansion ≥10 mm compared to baseline imaging.
2. Persistent Type II endoleak confirmed by CTA with ongoing sac perfusion.
3. Symptomatic presentation (e.g., abdominal or back pain) associated with aneurysm growth or endoleak.

### Preoperative morphological characteristics

All patients were screened based on the morphology of the aneurysm on CTA. Endosize (Therenva, France) and 3-Mensio Vascular (Pie Medical Imaging, Netherlands) software were used to measure the morphological characteristics. The measurement method followed the approach described by Gallitto et al. (10–12). The definitions of preoperative variables followed the standards of the Society for Vascular Surgery (SVS) and the International Society of Cardiovascular Surgery (ISCS) (13, 14). Aneurysm characteristics included neck features: neck angle (α), defined as the angle between the centerline of the suprarenal abdominal aorta and the centerline of the infrarenal abdominal aorta; neck angle (β), or the angle between the neck of the aneurysm and the centerline of the aneurysm body. Specialized vascular analysis software 3-Mensio Vascular was used to semi-automatically calculate aneurysm body volume (ABV), aneurysm thrombus volume (ATV), and ABV/ATV ratio (%VT) from the lowest renal artery to aortic bifurcation. EndoSize software was used to measure arterial diameters, and T2EL occurrence was determined based on CTA. No significant differences in T2EL rates were observed among the three device types (Medtronic Endurant: 12.8%, Cook Zenith: 13.0%, Gore Excluder: 14.1%; *P* = 0.34). Similarly, type Ia endoleak rates were low and comparable across devices (Endurant: 2.1%, Zenith: 1.6%, Excluder: 3.1%; *P* = 0.45).

### Statistical analysis

Descriptive analysis was performed to report the clinical characteristics and outcomes of the cohort. Numerical data were presented as frequencies or percentages for categorical factors and analyzed using chi-square tests or Mann–Whitney *U* tests. Continuous variables were analyzed using Student's *t*-test and expressed as mean ± standard deviation (SD). Receiver operating characteristic (ROC) curves were generated to calculate the area under the curve (AUC). Data analysis was conducted using R version 4.1.2 (https://www.r-project.org/).

## Results

### Characteristics of patients

The demographic characteristics and follow-up time of all patients are shown in Table 1. The T2EL-related reintervention group had a mean age of 69.74 ± 7.83 years, while the non-T2EL group had a mean age of 66.3 ± 8.05 years (*P* = 0.004). The incidence of hypertension was 56% in the T2EL-related reintervention group and 39.7% in the non-T2EL group (*P* = 0.03). There were no significant differences in other baseline characteristics between the two groups. The median time from T2EL intervention to the first surgery was 11 days (range: 83-5) in the T2EL-related reintervention group, while the median follow-up time based on the latest imaging was 12 days (range: 66-1) in the non-T2EL group.

**Table 1:** Basic data and follow-up information.

Items	T2EL group	Non-T2EL group	*t*/*χ*^2^/*z*	*P*
Age (years)	69.74 ± 7.83	66.3 ± 8.05	2.876	0.004
Gender (male)	90.00%	85.50%	0.722	0.395
Smoke	58.00%	55.25%	0.564	0.453
Hypertension	56.00%	39.7%	4.735	0.03
Coronary heart disease	38.00%	36.00%	0.075	0.784
Diabetes Mellitus	16.00%	12.31%	0.529	0.467
Cerebrovascular Disease	18.00%	29.69%	0.079	0.778
Pulmonary disease	12.00%	10.46%	0.108	0.743
Renal insufficiency	6.00%	2.50%	1.906	0.167
Peripheral vascular diseases	22.00%	12.30%	3.464	0.063
Length of stay (days)	15.36 ± 13.17	14.38 ± 8.29	−0.513	0.61

### Morphological characteristics of abdominal aortic aneurysm (AAA)

The morphological characteristics of the aneurysm neck and aneurysm sac are shown in Table 2. In terms of the morphology of the aneurysm neck, the mean maximum diameter in the T2EL-related reintervention group was 22.63 ± 3.46 mm, while it was 23.07 ± 12.66 mm in the non-T2EL-related reintervention group. The neck length was 25.73 ± 12.28 mm in the T2EL-related reintervention group and 31.91 ± 24.25 mm in the non-T2EL group. The neck angles (α) were 38.45 ± 26.66 and 33.16 ± 23.66 degrees in the T2EL-related reintervention group and non-T2EL group, respectively, while the neck angles (β) were 47.83 ± 23.46 and 51.65 ± 40.35 degrees, respectively. The maximum diameter of the aneurysm sac was 58.35 ± 13.07 mm in the T2EL-related reintervention group and 50.86 ± 14.70 mm in the non-T2EL group. The proportion of thrombus in the aneurysm sac was lower in the T2EL-related reintervention group (29.74 ± 9.86%) compared to the non-T2EL group (49.49 ± 13.027%) (*P* < 0.001).

**Table 2:** Morphological characteristics of aneurysms.

Items	T2EL group	Non-T2EL group	*t*/χ^2^ *z*	*P*
Maximum diameter (cm)	22.63 ± 3.46	23.07 ± 12.66	0.248	0.804
Neck length (cm)	25.73 ± 12.28	31.91 ± 24.25	1.768	0.078
Neck Angle (α°)	38.45 ± 26.66	33.16 ± 23.66	−1.449	0.148
Neck Angle (β°)	47.83 ± 23.46	51.65 ± 40.35	0.652	0.515
Tumor diameter (mm)	58.35 ± 13.07	50.86 ± 14.70	−3.403	<0.01
IMA diameter (mm)	3.17 ± 0.30	3.02 ± 0.34	−3.008	0.003
Thrombus ratio	29.74 ± 9.86	49.49 ± 13.02	12.577	<0.01
LA	5.98 ± 1.15	4.51 ± 1.41	−8.099	<0.01

### Intraoperative analysis

In terms of intraoperative conditions, the mean operative time for the initial EVAR procedure was 103.86 ± 19.03 min in the T2EL-related reintervention group and 99.84 ± 20.38 min in the non-T2EL-related reintervention group (*t* = −1.311, *P* = 0.191), indicating no significant difference between the two groups regarding the duration of their initial EVAR surgeries. The radiation dose was 631.38 ± 87.00 Gy in the T2EL-related reintervention group and 653.48 ± 83.89 Gy in the non-T2EL-related reintervention group (*t* = −1.726, *P* = 0.085), again with no significant difference. The mean blood loss was 46.80 ± 22.54 ml in the T2EL-related reintervention group and 49.35 ± 19.95 ml in the non-T2EL-related reintervention group (*t* = −0.828, *P* = 0.408), and there was no statistically significant difference between the two groups.

### Multivariable logistic analysis

Based on univariate analysis, six clinical variables, including maximum diameter of the aneurysm sac, proportion of intraluminal thrombus, diameter of the inferior mesenteric artery (IMA), number of patent lumbar arteries (LA), age, and hypertension, were selected as predictors of T2EL-related reintervention after EVAR. Logistic analysis showed that the maximum diameter of the aneurysm sac, IMA diameter, and number of patent LA were positively associated with the risk of T2EL-related reintervention, while the proportion of intraluminal thrombus was negatively associated with the risk (Table 3).

**Table 3:** Multifactorial analysis of risk factors that may lead to T2EL reintervention.

Items	T2EL group	Non-T2EL group	*t*/χ^2^/*z*	*P* *	Odds ratio (95% CI)	*P* **
Aneurysm diameter (mm)	58.35 ± 13.07	50.86 ± 14.70	−3.403	<0.01	1.043 (1.015,1.072)	0.002
IMA diameter (mm)	3.17 ± 0.30	3.02 ± 0.34	−3.008	0.003	3.901 (1.116,13.632)	0.033
Thrombus ratio	29.74 ± 9.86	49.49 ± 13.02	12.577	<0.01	0.895 (0.864,0.927)	<0.01
LA	5.98 ± 1.15	4.51 ± 1.41	−8.099	<0.01	2.548 (1.722,3.769)	<0.01
Patient age	69.74 ± 7.83	66.3 ± 8.05	2.876	0.004	0.962 (0.914,1.012)	0.138
Hypertension	56.00%	39.7%	4.735	0.03	0.442 (0.19,1.03)	0.058

* *P*, *t*/(2/*z*); Odds ratio.

** *P*, logistic regression analysis.

### Receiver operating characteristic (ROC) curve analysis

Based on the above analysis, the diameter of the aneurysm sac, proportion of intraluminal thrombus, IMA diameter, and number of patent LA were found to be potentially related to the need for reintervention after EVAR in T2EL patients. Therefore, ROC curves were constructed to determine the cutoff values for these variables associated with the need for reintervention.

The ROC curve indicated a cutoff value of 53.55 mm for the maximum diameter of the aneurysm sac (sensitivity 0.68, specificity 0.385) with an area under the curve (AUC) of 0.664. The cutoff value for the proportion of intraluminal thrombus was 42.50% (sensitivity 0.742, specificity 0.04) with an AUC of 0.91. The cutoff value for IMA diameter was 2.95 cm (sensitivity 0.78, specificity 0.505) with an AUC of 0.645. The cutoff value for the number of patent LA was 5.5 (sensitivity 0.72, specificity 0.305) with an AUC of 0.782.

**Figure 1:**
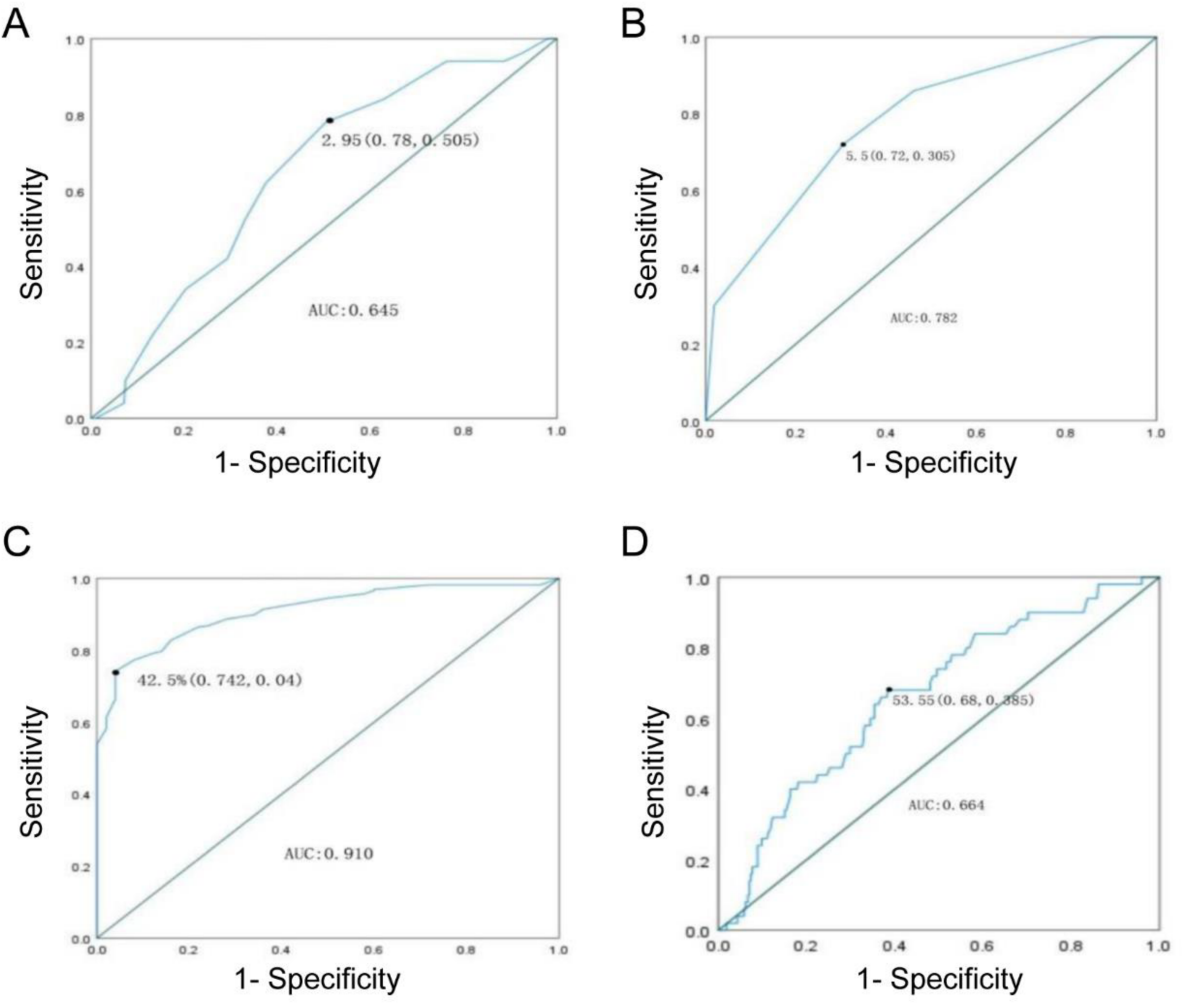
ROC curve. **(A)** IMA; **(B)** LA; **(C)** Thrombus ratio; **(D)** Maximum diameter.

The results suggest that patients with a maximum diameter of the aneurysm sac ≥53.55 mm, IMA diameter ≥2.95 mm, proportion of intraluminal thrombus ≤42.50%, and number of patent LA ≥5.5 have a higher risk of reintervention due to T2EL after EVAR (Figure 1).

## Discussion

This study aimed to analyze the risk factors associated with Type II endoleak (T2EL) requiring reintervention after endovascular aneurysm repair (EVAR) in patients with abdominal aortic aneurysms (AAAs). Our findings identified several key risk factors, including maximum aneurysm diameter, inferior mesenteric artery (IMA) diameter, number of patent lumbar arteries (LA), and proportion of intraluminal thrombus. These factors were significantly associated with the need for reintervention after EVAR.

The results of our study are consistent with previous research, which has also highlighted the importance of aneurysm sac diameter and the number of patent lumbar arteries as predictors of T2EL-related reintervention (6, 7, 15). Additionally, our findings suggest that the proportion of intraluminal thrombus may act as a protective factor against reintervention, which aligns with earlier studies (10).

In this study, the incidence of late T2EL at six months was 8.9%, which was notably lower than historical control data (16.3%) (15). Comparatively, the ENGAGE registry reported a five-year T2EL incidence of 15.6% (16), while U.S. regulatory trials reported a one-year T2EL incidence of 9.1% (17). This discrepancy may be attributed to racial differences, variations in coagulation function, and differences in study design (17, 18). Despite these variations, the T2EL incidence in our study remained lower than most historical controls, underscoring the effectiveness of P-TAE in preventing T2ELs.

The success rate of P-TAE in this study was 80.8%, with specific rates of 86.3% for the inferior mesenteric artery (IMA) and 80.3% for lumbar arteries (LA). These results align with those reported by Bishay et al. (19) and Fontana et al. (20), indicating high success rates across different studies, especially when performed by experienced interventional radiologists. Although the overall success rate was high, some patients, particularly those with complex anatomical structures (55.6%), were unable to achieve successful embolization, highlighting the need for technical improvements in certain cases.

Additionally, this study analyzed the risk of reintervention in T2EL patients, identifying critical thresholds of IMA diameter ≥2.95 mm and thrombus volume <42.5% as potential predictors of reintervention. These thresholds differ slightly from previously reported values, such as IMA diameter ≥2.5 mm (14) or ≥3.5 mm (21) and thrombus volume <40.0% (10), yet remain relevant. Through ROC curve analysis, four new thresholds were identified, potentially indicating the need for reintervention in T2EL patients. These thresholds can help assess the likelihood of reintervention post-EVAR and facilitate proactive interventions to prevent hospital readmission.

The devices used in this study, including the Medtronic Endurant, Cook Zenith, and Gore Excluder, demonstrated comparable rates of T2EL and type Ia endoleaks. These findings are consistent with previous studies, such as the ENGAGE registry, which reported a five-year T2EL incidence of 15.6% for the Endurant stent graft (16), and U.S. regulatory trials, which reported a one-year T2EL incidence of 9.1% for the same device (17). Similarly, studies involving the Cook Zenith and Gore Excluder devices have shown comparable outcomes, with T2EL rates ranging from 10%–15% (18, 19). These results suggest that the choice of endograft may have a limited impact on the incidence of T2EL, and that other factors, such as aneurysm morphology and patient characteristics, may play a more significant role in determining the need for reintervention.

Previous studies have identified several potential risk factors for T2EL occurrence after EVAR, including advanced age, chronic renal failure, chronic obstructive pulmonary disease (COPD), smoking, hypertension, and specific aneurysm anatomical characteristics (7, 18, 22). In our study, advanced age and hypertension were confirmed as independent risk factors for increased reintervention rates in T2EL patients. A large-scale prospective study conducted in Japan (7), which included medical records of 17,099 AAA patients treated with EVAR, demonstrated that age is an independent risk factor for T2EL, consistent with previous studies (6, 23) and our findings. Smoking and hypertension are also documented as significant risk factors for AAA development and progression. Quitting smoking has been shown to reduce the risk of AAA rupture by 20% (24). Furthermore, both the aforementioned prospective study (7), recent meta-analyses (6, 25), and retrospective studies (15, 26) suggest that smoking and hypertension may contribute to T2EL occurrence. Therefore, preventive measures, such as prophylactic endovascular embolization, should be considered for patients with T2EL-related risk factors before direct EVAR intervention.

T2ELs typically originate from the inferior mesenteric artery, lumbar arteries, and accessory renal arteries, which are the main branches of infrarenal AAAs. According to prior research, the inferior mesenteric artery is the largest branch, and diameters >2.5 mm (14, 23) or >3.5 mm (24) increase the risk of T2EL after EVAR. Additionally, an increased number of patent lumbar arteries may also elevate the risk of T2EL after EVAR. One study identified a threshold of 5.5 for lumbar arteries promoting T2EL after EVAR (20), consistent with our findings. Currently, it is widely accepted that the maximum diameter and number of supplying arteries are critical risk factors for T2EL. In our study, we found that the diameter and number of supplying arteries were independent risk factors for T2EL-related reintervention. The diameter of the inferior mesenteric artery in the T2EL reintervention group was larger, with a threshold of 2.95 mm defined by ROC curve analysis. Logically, larger supplying artery diameters make occlusion more challenging, leading to persistent blood supply between the stent graft and the aortic wall, thereby promoting T2EL formation. The ROC curve analysis demonstrated good sensitivity and specificity, supporting the conclusion that supplying artery diameter is a risk factor for T2EL-related reintervention. The number of patent lumbar arteries was another risk factor for reintervention. Recent studies (15, 27) indicate that a higher number of patent lumbar arteries (4–6) increases the risk of T2EL. Our univariate and multivariate analyses revealed significant statistical differences in the number of patent lumbar arteries between the T2EL reintervention group and the non-T2EL group, suggesting that patients with a higher number of patent lumbar arteries are more likely to experience T2EL-related reintervention after EVAR.

Reintervention was indicated in cases of aneurysm sac expansion ≥10 mm compared to baseline imaging [as recommended by the Society for Vascular Surgery (SVS) guidelines] (8), persistent Type II endoleak confirmed by CTA with ongoing sac perfusion [in line with the European Society for Vascular Surgery (ESVS) guidelines] (9), or symptomatic presentation (e.g., abdominal or back pain) associated with aneurysm growth or endoleak (consistent with both SVS and ESVS recommendations) (8, 9). These criteria emphasize the importance of monitoring sac expansion and persistent endoleaks as key indicators for reintervention, as they are strong predictors of aneurysm instability, rupture risk, and late complications (10, 15).

Interestingly, the presence of intraluminal thrombus in the aneurysm sac may act as a protective factor against T2EL-related reintervention. Previous studies indicate that patients with less than 40% intraluminal thrombus often require reintervention after EVAR (10). However, earlier research on AAA pathogenesis classified intraluminal thrombus as a negative factor, potentially exacerbating oxidative stress and promoting inflammatory mediator recruitment, thus contributing to AAA progression (24). Consequently, the exact role of intraluminal thrombus in AAA remains unclear. In our study, the proportion of intraluminal thrombus was lower in the T2EL group than in the non-T2EL group, with a threshold of 42.5% identified through ROC curve analysis, indicating a protective effect against reintervention. The presence of intraluminal thrombus facilitates branch occlusion and enhances the seal between the stent graft and the arterial wall, reducing the likelihood of T2EL-related reintervention. Therefore, preventive embolization of the aneurysm sac during surgery to increase the proportion of intraluminal thrombus could play a positive role in preventing T2EL-related reintervention, particularly in patients with risk factors.

### Insufficient research

This study has some limitations. Firstly, it is a small-sample, retrospective study conducted at three centers. It is necessary to conduct prospective studies in larger treatment centers to confirm the findings of this study. Additionally, due to the limited number of patients, we did not differentiate between early and late type II endoleaks (T2EL), nor did we consider the impact of risk factors on different stages of T2EL. Future research can be conducted on larger cohorts to address these issues. Lastly, we did not analyze the relationship between the use of antiplatelet medications, hemodynamic characteristics, and the possibility of T2EL-related reinterventions, which should be explored in future studies.

## Conclusion

Endovascular aneurysm repair (EVAR) is a safe and feasible treatment option for most patients with abdominal aortic aneurysms (AAA). However, accurate prediction and intervention of postoperative T2EL and associated reinterventions are still needed. Through this study, by understanding the risk factors associated with T2EL-related reinterventions, operators can more accurately estimate the risk of T2EL occurrence after EVAR and take appropriate preventive measures such as prophylactic embolization of the aneurysm sac during the EVAR procedure based on the assessment results.

## Data Availability

The original contributions presented in the study are included in the article/Supplementary Material, further inquiries can be directed to the corresponding authors.

## References

[1] Wanhainen A Verzini F Van Herzeele I Allaire E Bown M Cohnert T Editor’s choice—European society for vascular surgery (ESVS) 2019 clinical practice guidelines on the management of abdominal aorto-iliac artery aneurysms. Eur J Vasc Endovasc. (2019) 57(1):8–93. 10.1016/j.ejvs.2018.09.020 30528142

[2] Ullery BW Lee JT Dalman RL. Snorkel/chimney and fenestrated endografts for complex abdominal aortic aneurysms. J Cardiovasc Surg. (2015) 56(5):707–17. 25800354

[3] Pena CS Schiro BJ Benenati JF. Fenestrated endovascular abdominal aortic aneurysm repair. Tech Vasc Interv Rad. (2018) 21(3):156–64. 10.1053/j.tvir.2018.06.005 30497550

[4] White GH Yu W May J. Endoleak–a proposed new terminology to describe incomplete aneurysm exclusion by an endoluminal graft. J Endovasc Surg. (1996) 3(1):124–5. 10.1583/1074-6218(1996)003<0124b:>2.0.CO;2 8991758

[5] Ameli-Renani S Pavlidis V Morgan RA. Secondary endoleak management following TEVAR and EVAR. Cardiovasc Inter Rad. (2020) 43(12):1839–54. 10.1007/s00270-020-02572-9 PMC7649162 32778905

[6] Sidloff DA Stather PW Choke E Bown MJ Sayers RD. Type II endoleak after endovascular aneurysm repair. Brit J Surg. (2013) 100(10):1262–70. 10.1002/bjs.9181 23939840

[7] Seike Y Matsuda H Shimizu H Ishimaru S Hoshina K Michihata N Nationwide analysis of persistent type II endoleak and late outcomes of endovascular abdominal aortic aneurysm repair in Japan: a propensity-matched analysis. Circulation. (2022) 145(14):1056–66. 10.1161/CIRCULATIONAHA.121.056581 35209732 PMC8969842

[8] Chaikof EL Dalman RL Eskandari MK Jackson BM Lee WA Mansour MA The society for vascular surgery practice guidelines on the care of patients with an abdominal aortic aneurysm. J Vasc Surg. (2018) 67(1):2–77. 10.1016/j.jvs.2017.10.044 29268916

[9] Sidloff DA Gokani V Stather PW Choke E Bown MJ Sayers RD. Type II endoleak: conservative management is a safe strategy. Eur J Vasc Endovasc. (2014) 48(4):391–9. 10.1016/j.ejvs.2014.06.035 25042332

[10] Gallitto E Gargiulo M Mascoli C Freyrie A DE Matteis M Serra C Persistent type II endoleak after EVAR: the predictive value of the AAA thrombus volume. J Cardiovasc Surg. (2018) 59(1):79–86. 10.23736/S0021-9509.16.08842-X 26221867

[11] Piazza M Squizzato F Miccoli T Lepidi S Menegolo M Grego F Definition of type II endoleak risk based on preoperative anatomical characteristics. J Endovasc Ther. (2017) 24(4):566–72. 10.1177/1526602817712511 28578623

[12] Chaikof EL Blankensteijn JD Harris PL White GH Zarins CK Bernhard VM Reporting standards for endovascular aortic aneurysm repair. J Vasc Surg. (2002) 35(5):1048–60. 10.1067/mva.2002.123763 12021727

[13] Chaikof EL Fillinger MF Matsumura JS Rutherford RB White GH Blankensteijn JD Identifying and grading factors that modify the outcome of endovascular aortic aneurysm repair. J Vasc Surg. (2002) 35(5):1061–6. 10.1067/mva.2002.123991 12021728

[14] Fukuda T Matsuda H Sanda Y Morita Y Minatoya K Kobayashi J CT findings of risk factors for persistent type II endoleak from Inferior mesenteric artery to determine indicators of preoperative IMA embolization. Ann Vasc Dis. (2014) 7(3):274–9. 10.3400/avd.oa.14-00008 25298829 PMC4180689

[15] Meshii K Sugimoto M Niimi K Kodama A Banno H Komori K. The association between perioperative embolization of hypogastric arteries and type II endoleaks after endovascular aortic aneurysm repair. J Vasc Surg. (2021) 73(1):99–107. 10.1016/j.jvs.2020.04.505 32442614

[16] Teijink J Power AH Bockler D Peeters P van Sterkenburg S Bouwman LH Editor’s choice—five year outcomes of the endurant stent graft for endovascular abdominal aortic aneurysm repair in the ENGAGE registry. Eur J Vasc Endovasc. (2019) 58(2):175–81. 10.1016/j.ejvs.2019.01.008 31235305

[17] Singh MJ Fairman R Anain P Jordan WD Maldonado T Samson R Final results of the endurant stent graft system in the United States regulatory trial. J Vasc Surg. (2016) 64(1):55–62. 10.1016/j.jvs.2015.12.048 27131927

[18] Shalaby SY Foster TR Hall MR Brownson KE Vasilas P Federman DG Systemic inflammatory disease and its association with type II endoleak and late interventions after endovascular aneurysm repair. Jama Surg. (2016) 151(2):147–53. 10.1001/jamasurg.2015.3219 26501863

[19] Bishay VL Biederman DM Ward TJ van der Bom IM Patel RS Kim E Transradial approach for hepatic radioembolization: initial results and technique. Am J Roentgenol. (2016) 207(5):1112–21. 10.2214/AJR.15.15615 27767350

[20] Fontana F Piacentino F Ossola C Coppola A Curti M Macchi E Transcatheter arterial embolization in acute non-variceal gastrointestinal bleedings: a ten-year single-center experience in 91 patients and review of the literature. J Clin Med. (2021) 10(21):4979. 10.3390/jcm10214979 34768505 PMC8584454

[21] Suarez GL Lozano MI Montoya CN Fernandez-Samos GR Vallina-Victorero VM. Preoperative predictive factors for type II endoleak: trying to define high-risk patients. Asian J Surg. (2023) 46(1):187–91. 10.1016/j.asjsur.2022.03.022 35317967

[22] Samura M Morikage N Otsuka R Mizoguchi T Takeuchi Y Nagase T Endovascular aneurysm repair with Inferior mesenteric artery embolization for preventing type II endoleak: a prospective randomized controlled trial. Ann Surg. (2020) 271(2):238–44. 10.1097/SLA.0000000000003299 30946077

[23] Deery SE Ergul EA Schermerhorn ML Siracuse JJ Schanzer A Goodney PP Aneurysm sac expansion is independently associated with late mortality in patients treated with endovascular aneurysm repair. J Vasc Surg. (2018) 67(1):157–64. 10.1016/j.jvs.2017.06.075 28865980 PMC6114145

[24] Golledge J. Abdominal aortic aneurysm: update on pathogenesis and medical treatments. Nat Rev Cardiol. (2019) 16(4):225–42. 10.1038/s41569-018-0114-9 30443031

[25] Charisis N Bouris V Conway AM Labropoulos N. A systematic review and pooled meta-analysis on the incidence and temporal occurrence of type II endoleak following an abdominal aortic aneurysm repair. Ann Vasc Surg. (2021) 75:406–19. 10.1016/j.avsg.2021.01.083 33549794

[26] Ide T Masada K Kuratani T Sakaniwa R Shimamura K Kin K Risk analysis of aneurysm sac enlargement caused by type II endoleak after endovascular aortic repair. Ann Vasc Surg. (2021) 77:208–16. 10.1016/j.avsg.2021.06.013 34461238

[27] Lo RC Buck DB Herrmann J Hamdan AD Wyers M Patel VI Risk factors and consequences of persistent type II endoleaks. J Vasc Surg. (2016) 63(4):895–901. 10.1016/j.jvs.2015.10.088 26796291 PMC4808613

